# Chaotic advection mixer for capturing transient states of diverse biological macromolecular systems with time-resolved small-angle X-ray scattering

**DOI:** 10.1107/S2052252523003482

**Published:** 2023-04-28

**Authors:** Kara A. Zielinski, Andrea M. Katz, George D. Calvey, Suzette A. Pabit, Shawn K. Milano, Cody Aplin, Josue San Emeterio, Richard A. Cerione, Lois Pollack

**Affiliations:** a School of Applied and Engineering Physics, Cornell University, Ithaca, New York USA; b Department of Chemistry and Chemical Biology, Cornell University, Ithaca, New York USA; c Department of Molecular Medicine, Cornell University, Ithaca, New York USA; UCL, United Kingdom

**Keywords:** mix-and-inject techniques, time-resolved SAXS, structural biology, biomacromolecular systems

## Abstract

Advances in time-resolved structural techniques, mainly in macromolecular crystallography and small-angle X-ray scattering (SAXS), allow for a detailed view of the dynamics of biological macromolecules and reactions between binding partners. Of particular promise, are mix-and-inject techniques, which offer a wide range of experimental possibility as microfluidic mixers are used to rapidly combine two species just prior to data collection. Most mix-and-inject approaches rely on diffusive mixers, which have been effectively used within crystallography and SAXS for a variety of systems, but their success is dependent on a specific set of conditions to facilitate fast diffusion for mixing. The use of a new chaotic advection mixer designed for microfluidic applications helps to further broaden the types of systems compatible with time-resolved mixing experiments. The chaotic advection mixer can create ultra-thin, alternating layers of liquid, enabling faster diffusion so that even more slowly diffusing molecules, like proteins or nucleic acids, can achieve fast mixing on timescales relevant to biological reactions. This mixer was first used in UV–vis absorbance and SAXS experiments with systems of a variety of molecular weights, and thus diffusion speeds. Careful effort was also dedicated to making a loop-loading sample-delivery system that consumes as little sample as possible, enabling the study of precious, laboratory-purified samples. The combination of the versatile mixer with low sample consumption opens the door to many new applications for mix-and-inject studies.

## Abstract 2

A chaotic advection mixer is employed to expand the range of reactions that can be probed with time-resolved solution scattering.

## 1. Introductions

Biological macromolecules are dynamic molecular machines that perform functions essential to life. Much insight into their function has been gained from probing their structures, dynamics and interactions with binding partners. Time-resolved techniques for measuring structures are a powerful tool for observing molecular interactions, providing insights into both the underlying principles and the development of therapeutics to modify these interactions. Reactions must be rapidly initiated, and subsequently probed as they progress at different time points. Many structural probes have been employed to capture transient structures. These include crystallography (Moffat, 1998 ▸; Olmos *et al.*, 2018 ▸; Pandey *et al.*, 2021 ▸), spectroscopies (van Nuland *et al.*, 1998 ▸; Calvey *et al.*, 2020 ▸), small-angle X-ray scattering (SAXS) (Plaxco & Dobson, 1996 ▸; Pollack *et al.*, 1999 ▸; Plumridge *et al.*, 2018 ▸) and cryo-electron microscopy (Lu *et al.*, 2009 ▸; Frank, 2017 ▸).

SAXS is particularly well suited for time-resolved studies, reporting the overall size, shape and degree of compactness of dynamic ensembles of molecules in solution at room temperature (Svergun & Koch, 2003 ▸). Its solution nature provides a distinct advantage over other methods that require constraints, such as crystallization, labeling, the presence of spectroscopically active species or freezing. SAXS probes molecules ranging in size from a few kilodaltons to gigadaltons (Kikhney & Svergun, 2015 ▸), is sensitive to intermolecular interactions and molecular weight, and can provide structural information on the tens of Ångstrom scale. Other light scattering techniques, such as dynamic light scattering (DLS), can provide similar information, but at much lower resolution. In addition, time-resolved SAXS (TR-SAXS) can detect the structures of transient interactions that seed the formation of larger complexes. At the concentrations required for many structural studies, samples may form higher order assemblies in equilibrium, and in some cases, this process is biologically relevant and can more closely mimic cellular environments. The ability to monitor the earliest structural intermediates (*e.g.* those resulting from bi-molecular interactions that precede large scale association), provides unique information about how complexes form. These first steps can offer novel insight into how large structures assemble, and can be directed at either ordered structures such as viruses (Khaykelson & Raviv, 2020 ▸) or disordered structures that arise, *e.g.* from liquid–liquid phase separation (Martin *et al.*, 2021 ▸).

The two broad categories of TR-SAXS experiments are distinguished by the reaction trigger used: light-activated or chemically triggered processes. Light can trigger photoisomerization, a temperature jump or the release of a caged compound (Cho *et al.*, 2021 ▸; Thompson *et al.*, 2019 ▸; Monteiro *et al.*, 2021 ▸). However, most biological macromolecules are not light-activated, limiting the reach of this trigger. Chemically triggered processes, enabled by rapid mixing of solutions, can be applied to a broader range of systems. Previous SAXS mixing experiments utilized stopped-, continuous- or turbulent-flow mixers (Pollack & Doniach, 2009 ▸; Graceffa *et al.*, 2013 ▸). In a stopped-flow experiment, two solutions are rapidly mixed and injected into an observation chamber. The flow is stopped, trapping the reacting sample in the observation chamber. Measurements of the stationary sample are carried out as the mixture ages, in real time. To reach the shortest points, rapid exposure is required, resulting in noisy data, and the need to repeat the experiment many times to accumulate good statistics. Stopped-flow mixers are sample-intensive, using hundreds of microlitres of sample per shot (Panine *et al.*, 2006 ▸). Radiation damage may occur when measuring longer time points, forcing an attenuation of the X-ray beam. In continuous-flow mixing, two solutions are rapidly mixed, then travel down an observation channel to allow the reaction to progress for a set amount of time before encountering the X-ray beam. Many of these mixers exploit diffusion via flow-focusing, which utilizes a second co-flowing fluid stream to thin the central, sample-containing stream. Small molecules contained in the sheath rapidly diffuse into the sample stream, initiating the reaction. These mixers consume far less sample than stopped-flow, but the thin ribbon of sample requires a small X-ray beam and longer acquisition times to acquire high signal-to-noise data (Pollack *et al.*, 2001 ▸; Lamb *et al.*, 2008 ▸) unless an expanded observation region is present (Plumridge *et al.*, 2018 ▸). For optimal performance, the reactant molecule should be small (*e.g.* quickly diffusing) and highly soluble. These constraints preclude the study of some small-molecule drug targets, which are typically hydro­phobic, have low solubility or are unavailable in large quantities. It can also be difficult, with a diffusive mixer, to achieve one-to-one mixing ratios or to probe concentration-sensitive reactions; the concentration of the diffusing reactant increases along the detection channel. Rapid reactions between macromolecular species (*e.g.* protein–protein or protein–nucleic acid interactions), or in viscous media, can be limited by slower diffusion times. Under some circumstances, mixing times can exceed the timescales of interest for the reaction (approximately milliseconds to single seconds). Turbulent mixers circumvent some of the above challenges. They eliminate the need for a thin sample stream, produce higher signal-to-noise data in shorter collection times and allow study of a broader range of reactions. Their drawback is the high sample consumption, as very high sample flow rates are required for efficient mixing. Instrumentation advances significantly reduced the sample consumed by a turbulent SAXS mixer to 2–3 mg per time point (Graceffa *et al.*, 2013 ▸), but this quantity may still be prohibitive for some biological samples.

**Figure 1:**
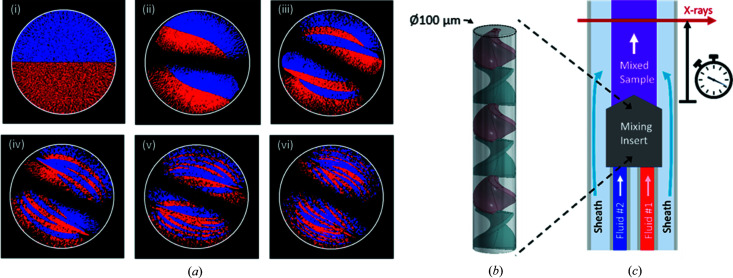
Simulation (ANSYS Fluent 18.2, ANSYS, Inc. Canonsburg, Pennsylvania) and conceptional design of the Kenics mixer. (*a*) Cross-sectional view of the simulated flows at different locations inside a Kenics mixer with a twist angle of 135° and an aspect ratio of 1.125. Locations are as follows: (i) immediately before the flows encounter the first element, (ii) after one helical element, (iii) after two elements, (iv) after three elements, (v) after four elements, (vi) after five elements. These images have been smoothed to increase the apparent size of the streamlines to make them easier to see. This may reduce or eliminate the visibility of thinner striations in the later panels. (*b*) Kenics mixer. The cylindrical pipe through which liquid flows is drawn in transparent gray. (*c*) Overall device concept. A mixing insert (gray) combines two fluids (red and blue). The mixed sample (purple) flows out of the insert and is surrounded by a sheath flow (light blue). X-rays probe the mixed sample after a pre-determined delay.

A chaotic advection mixer, also known as a static mixer, bypasses all the above-described limitations. These mixers consist of a fluidic channel containing carefully arranged geometric elements that induce mixing via a process known as a baker’s transformation (Fig. 1 ▸). As two fluids travel down the channel, the elements stretch the interfaces between the fluids, split them apart and then stack them to create thinner layers with more interfaces (Saatdjian *et al.*, 2012 ▸; Wiggins & Ottino, 2004 ▸). Several baker’s transformations occur as the fluids traverse the static mixer, so that many alternating thin ‘strips’ of each fluid are formed. Static mixers are used in industry, and some designs can be scaled down and incorporated into microfluidic channels for efficient, laminar flow mixing of small volumes (Knoška *et al.*, 2020 ▸; Bertsch *et al.*, 2001 ▸). The production of thin layers of fluid via the baker’s transformation is key to efficient mixing as even large macromolecules, or those in viscous media, can diffuse rapidly to mix across these very small-length scales. These shorter length scales also yield tighter reaction initiation, and complete mixing. Because concentration-sensitive reactions require a fixed ratio of reactant to sample, they can be readily performed in this platform. The chaotic advection mixer uses significantly less sample than a turbulent mixer, and its continuous-flow nature greatly reduces SAXS radiation damage relative to a stopped-flow mixer, making it an extremely unique and versatile option for performing time-resolved measurements.

Here, we present a novel application of a chaotic advection mixer with a custom sample environment that further reduces sample consumption. This system is optimized for efficient and effective acquisition of TR-SAXS data and includes a high-throughput loop-loading sample delivery system. Using a scaled-down version of an industrial static mixer, a Kenics design, this device produces high signal-to-noise data using only 1/10 of the sample required per time point by the most efficient turbulent SAXS mixer (Graceffa *et al.*, 2013 ▸), yet retains the ability to study a diverse range of biological interactions, regardless of biomacromolecule size, solubility or viscosity. We describe the mixer design, construction and use, and highlight applications to a broad range of biological systems to demonstrate the efficacy of this method in probing protein–protein associations (trypsin and aprotinin), protein–nucleic acid binding (GAC rRNA and L11 protein), Mg^2+^-induced RNA folding, concentration-sensitive reactions and its ability to observe molecules pre-aggregation (tissue transglutaminase with calcium), demonstrating all the applications introduced above.

## 2. Materials and methods

### 2.1. Sample preparation

Horse heart myoglobin was purchased from Sigma–Aldrich (M1882, T8003, A1153, St Louis, MO) and was dissolved in 100 m*M* sodium phosphate, pH 7.8, filtered to remove aggregates and impurities, and adjusted to a concentration of 1.5 m*M*. A stock solution of 1% sodium azide (786–750, G-Biosciences, St Louis, MO) was diluted with distilled water to produce concentrations ranging from 15 to 50 m*M* sodium azide.

Trypsin from bovine pancreas and aprotinin from bovine lung were purchased as lyophilized powders (T1426 and A1153, Sigma–Aldrich, St Louis, MO) and were separately dissolved in 20 m*M* Tris buffer, pH 7.0 with 40 m*M* KCl and 20 m*M* CaCl_2_. Protein concentrations were assessed from absorption measurements at 280 nm using extinction coefficients of 36 600 *M* cm^−1^ for trypsin and 3840 *M* cm^−1^ for aprotinin. For mixing experiments, aprotinin at 270 m*M* (1.73 mg ml^−1^) was chosen to be in molar excess of trypsin. A trypsin concentration of 170 m*M* (4 mg ml^−1^) was chosen for these experiments as a compromise between signal strength and avoiding interparticle interference effects.

The 58-nucleotide GAC ribosomal RNA from *E. coli* with the U1061A mutation (Grilley *et al.*, 2007 ▸) was transcribed from PCR-amplified DNA plasmid using a T7 polymerase kit (PROMEGA, Madison, WI). The RNA was purified from the T7 product using a Superdex 200 Increase 10 × 300 size-exclusion column (GE Healthcare, Chicago, IL) in 100 m*M* KCl and 10 m*M* Na-MOPSO buffer, pH 6.5. The RNA was concentrated in the same buffer and annealed at 65°C for 30 min, slowly cooled to room temperature and stored at 4°C prior to SAXS measurements. The RNA concentration was determined from absorption measurements at 260 nm, using the extinction coefficient 594 200 *M* cm^−1^. For folding experiments, 60–80 m*M* RNA was mixed with 10 m*M* MgCl_2_ in the same buffer. For binding experiments, 60–80 m*M* RNA was mixed with 80–100 m*M* L11 protein with 10 m*M* MgCl_2_ in the same buffer.

The 147-residue full-length L11 ribosomal protein from *T. thermophilus* was purified as described previously (Triantafillidou *et al.*, 1999 ▸). In brief, BL21(DE3) *E. coli* cells were transformed with His-tagged L11 recombinant plasmid with a thrombin cleavage site (pD431-SR/TthL11, made by DNA 2.0, Newark, CA). The cells were grown at 37°C until an optical density (OD) of 0.6 was reached. The expression of L11 protein was induced by the addition of 1 m*M* IPTG to the growth medium. To harvest the protein, the cells were centrifuged 4 h after induction and the pellets were collected, reconstituted and lysed using an Emulsiflex cell disruptor. The resulting crude lysate was passed through a Co^2+^ column (HiTrap Talon crude, GE Healthcare, Chicago, IL) and the Histidine-tag was cleaved using a thrombin cleavage kit (Thrombin CleanCleave Kit, Sigma–Aldrich, St Louis, MO). The efficiency of the cleavage and the purity of the protein were assessed using an SDS–PAGE. L11 protein was buffer-exchanged into 100 m*M* KCl, 10 m*M* Na-MOPSO buffer, pH 6.5, prior to SAXS measurements. The protein concentration was determined from absorbance at 280 nm, using the extinction coefficient 8480 *M* cm^−1^.

Recombinant tissue transglutaminase (tG) was expressed and purified as previously described (Datta *et al.*, 2006 ▸). Briefly, *E. coli* BL21 (DE3) competent cells (New England Biolabs) were transformed with a pET28a vector encoding human tG with a N-terminal six-histidine tag. The *E. coli* cells were grown in Luria broth (LB) with 50 µg ml^−1^ kanamycin at 37°C to an OD_600_ of 0.6–0.8, and protein expression was induced at 25°C with 10 µM IPTG for 16–18 h. The cells were resuspended in lysis buffer [50 m*M* Tris pH 8.5, 500 m*M* NaCl, 0.1 m*M* PMSF, 5 m*M* β-mercapto­ethanol (BME), 10%(*w*/*v*) glycerol, 50 µ*M* GTP] and disrupted by sonication. The lysate was clarified by centrifugation at 185 000*g* for 45 min. The supernatant was then loaded onto a 5 ml HisTrap HP column (Cytiva Life Sciences) equilibrated with equilibration buffer [50 m*M* Tris pH 8.5, 500 m*M* NaCl, 10%(*w*/*v*) glycerol, 0.1 m*M* PMSF, 5 m*M* BME], washed with 100 ml of equilibration buffer and then washed again with 100 ml of wash buffer [50 m*M* Tris pH 8.5, 10 m*M* NaCl, 10%(*w*/*v*) glycerol, 5 m*M* BME]. The protein was isocratically eluted with 50 ml of wash buffer with 320 m*M* imidazole. The eluent was loaded onto a 5 ml HiTrap Q HP column (Cytiva Life Sciences) and eluted with a gradient of Buffer A [50 m*M* MES pH 6.5, 10 m*M* NaCl, 10%(*w*/*v*) glycerol, 1 m*M* DTT] and Buffer B (Buffer A with 800 m*M* NaCl). The peak fractions were then pooled and loaded again onto a 5 ml HisTrap column equilibrated with HisTrap Buffer A [20 m*M* HEPES pH 7.5, 100 m*M* NaCl, 10%(*w*/*v*) glycerol], washed with 100 ml of HisTrap Buffer A, then eluted over a gradient with HisTrap Buffer B (HisTrap Buffer A with 500 m*M* imidazole). The peak fraction was then pooled and injected onto a HiLoad Superdex 200 column (Cytiva Life Sciences) equilibrated with tG gel filtration buffer [20 m*M* HEPES pH 7.5, 100 m*M* NaCl, 10%(*w*/*v*) glycerol, 1 m*M* DTT] for purification by size-exclusion chromatography. The purified tG was concentrated to 2 mg ml^−1^ using a 10 kDa cutoff centrifugal filter (Jumbosep, PALL), flash frozen in liquid nitro­gen and stored at −80°C. For time-resolved experiments, 2 mg ml^−1^ tG was mixed with 4 m*M* CaCl_2_ in the same gel-filtration buffer.

### 2.2. Time-resolved absorbance measurements for Kenics mixer characterization

Time-resolved absorbance experiments were performed to verify mixing using 560 ± 2 nm light and a Zyla CMOS camera (ANDOR, Oxford Instruments, Abingdon, UK) to illuminate and image the sample cell. The microscope was configured so that the field of view of the camera was several millimetres, therefore one image captured the reaction across a range of time points. Calibrations were performed using a well characterized reaction: myoglobin plus sodium azide (Marcoline & Elgren, 1998 ▸; Nami *et al.*, 2016 ▸).

PHD 4400 syringe pumps (Harvard Apparatus, Holliston, MA) were used to drive reacting solutions into the supply lines at 55.5 µl min^−1^ each. The sheath solution, driven at 189 µl min^−1^ by an OB1 pressure controller with feedback from an MFS flow sensor (Elveflow, Paris, France), contained sodium azide at the final mixed concentration to maintain a constant sodium azide concentration in the sample cell. These flow rates were chosen so that the camera could image a 10–90 ms reaction interval with a mixed sample path length of 250 µm. The reaction was probed at three sodium azide concentrations: 15, 20 and 25 m*M*, with both Kenics mixer designs. Data were also acquired when mixing with water for use as a reference. Dark counts were subtracted from all images. For each dataset, the transmission at each pixel was calculated as a ratio of the reacted data over the reference data, and then converted to absorbance. Flow velocities were used to convert the distance from the Kenics mixer to a time point. This yielded the final dataset of absorbance versus time that was used for kinetics modeling.

### 2.3. SAXS experiments

#### 2.3.1. Beamline requirements

All samples were characterized by static, equilibrium SAXS to find the optimal conditions. GAC rRNA, L11, trypsin and aprotinin were screened at the CHESS beamline ID7A1. For tG, SAXS conditions were screened using a home source (BioXolver, Xenocs Inc. Holyoke, MA). Time-resolved SAXS experiments were performed at the CHESS beamline ID7A1. X-ray energies of 11.3 keV were used. The beam size was maintained at 120 × 150 µm using custom-made single-crystal Ge slits inspired by Li *et al.* (2008 ▸). Normalization of scattered intensities was achieved using a semi-transparent beam stop made of 250 micrometre-thick molybdenum foil, which can block the direct beam to prevent detector damage while still providing a measure of transmitted counts for normalization (Kučerka *et al.*, 2008 ▸).

#### 2.3.2. Sample-delivery setup

The sample-delivery system must efficiently deliver all reactants to the mixer, allow easy switching between biomolecules and buffers for background subtraction with minimal dead volume, and maintain fast flow rate stabilization to conserve sample. These criteria are met with a loop-loading system, in which each species, sample or buffer, is loaded into two sets of separate loops (four loops total), connected to each of the two sides of the mixer. The loop volume was 75 µl for the sample and 150 µl for the buffer. Two high-pressure syringe pumps (PHD 4400, Harvard Apparatus, Holliston, MA) were used to drive syringes of inert mineral oil to push the species out of the loops and into the mixer. Two sets (one for each inlet port of the Kenics) of three valves – two high-pressure switching valves (Rheodyne MXP7970, IDEX Health and Science, West Henrietta, NY) and one injection valve (VICI Valco Instruments Co. Inc. Houston, TX) – were used to control this process (see the supporting information for more details and benefits). This setup also prevented the oil from entering the sample cell. Another injection valve (Rheodyne MXP7970, IDEX Health and Science, West Henrietta, NY) was used for automatic oil refilling of the syringes. An automatic cleaning station, with soap and water reservoirs pressurized with nitro­gen gas, was used to remove the oil, and clean and dry the loops so that they were ready for the next sample. To increase efficiency, the sample collection and cleaning was automated through customized software that remotely controls all pumps and valves. The supply lines connected directly to the mixer have inner diameters of 75–100 µm to reduce dead volumes and to keep the back pressure compatible with the range of the syringe pumps. More details of our sample-delivery setup can be found in Fig. S1 of the supporting information.

A sheath flow, which surrounds the mixed sample, was driven by a multichannel pressure controller (OB1, 0–8000 mbar range, Elveflow, Paris, France) and measured with flow sensors (MFS3 or MFS4 depending on flow conditions, Elveflow, Paris, France). The flow through the waste line of the device was monitored by a mass flow sensor (ML120V00 Mini Cori-flow, Bronkhorst USA Inc, Bethlehem, PA) which, in combination with the upstream MFS flow sensor, was used to monitor the total flow rate through the sample cell and ensure that the correct conditions were met while acquiring data. A schematic of the flow setup and operation protocols is provided in Fig. S1.

#### 2.3.3. Data analysis

Scattering images from samples and buffer (without samples) were collected and analyzed using *BioXTAS RAW* software (Hopkins *et al.*, 2017 ▸). For each measurement, datasets were comprised of 10–20 5 s frames. To extract the signal from the biomolecules alone, SAXS measurements of only the buffer were subtracted from the SAXS profiles that contained the molecular sample(s). Guinier fits were used to calculate the radius of gyration (*R*_g_) values, except for the trypsin and aprotinin series, which utilized *P*(*r*) instead due to small amounts of aggregation present in both samples before the reaction. Further data processing was performed using in-house MATLAB scripts.

## 3. Results

### 3.1. Chaotic advection mixer concept, design, fabrication, operation and testing

#### 3.1.1. Mixer concept

As described in the Introduction[Sec sec1], our goal is to optimize a static mixer for TR-SAXS experiments. One of the more easily adaptable static mixers is based on the Kenics design (Chemineer, Dayton, Ohio), shown in Fig. 1 ▸(*b*). Short, helical elements with alternating left- or right-handedness induce baker’s transformations [Fig. 1 ▸(*a*)] as the flow passes through them (Bertsch *et al.*, 2001 ▸; Galaktionov *et al.*, 2003 ▸; Hobbs & Muzzio, 1997 ▸). The aspect ratio, twist angle and number of helices can be optimized to mix the fluids of interest (Galaktionov *et al.*, 2003 ▸; Szalai & Muzzio, 2003 ▸); we selected a twist angle of 135° and an aspect ratio of 1.125 based on our simulations (ANSYS Fluent 18.2, ANSYS, Inc. Canonsburg, Pennsylvania). After passage through the Kenics element, the solution flows along a tube, where the distance traveled corresponds to the time point measured. Fig. 1 ▸(*a*) shows a simulation of the baker’s transformation at various points throughout the first five helical elements, showing the expected 2^*n*+1^ layers after *n* blades with the ‘strip’ pattern that agrees with simulations in the literature.

There are several important considerations when interfacing this design with X-rays. First, once the solution emerges from the mixing region, it should be fully sheathed in (surrounded by) water or buffer to keep the sample off the walls of the observation channel [Fig. 1 ▸(*c*)]. This is important because sample flowing near the walls is susceptible to radiation damage (Kirby *et al.*, 2016 ▸). Constraining the sample to the center of the channel also reduces the timing dispersion resulting from the parabolic flow profile. If a small ligand or ion is mixed and has a large enough diffusion coefficient (on the order of 10^−9^ m^2^ s^−1^) to diffuse appreciably in the radial direction of the sample cell, the concentration of the ligand can be kept constant in the sample cell by including the ligand in the sheath flow. Second, the cross section of the mixed fluid in the X-ray observation region should be large enough to accommodate the X-ray beam and to generate a strong scattering signal. Third, the tube that comprises the observation region should be made from X-ray transparent material with low intrinsic scatter. Finally, the diameter of the observation region should be such that a good compromise is made between X-ray signal strength (larger diameter) and reasonable timing uncertainty due to sample transit times through the beam (smaller diameter). Fig. 1 ▸(*c*) shows a conceptual drawing of a device that has all these desired qualities.

#### 3.1.2. Mixer design

**Figure 2:**
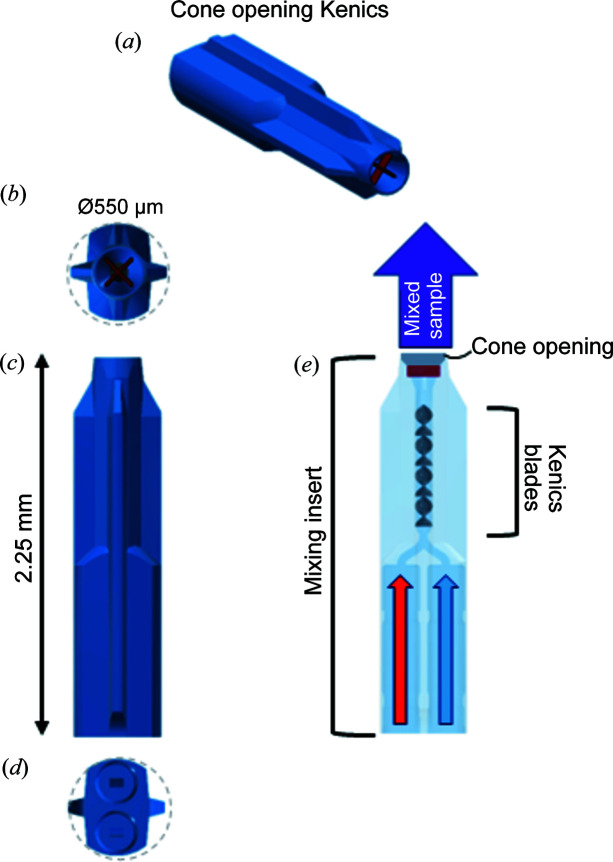
CAD-rendering of the 3D-printed mixing insert that houses the Kenics mixer. (*a*) Exterior of the device. (*b*) View of the cone opening from downstream, with the flow homogenizer shown in red. The dashed gray line represents the observation tube. (*c*) Side view of the cone opening insert. (*d*) View of the cone opening insert from upstream, showing the supply line ports. (*e*) Cross-sectional view of the cone opening insert with the cone opening shaded in gray.

Mixing inserts, which house the helical elements of the Kenics and include ports for the capillaries, were designed in *Autodesk Inventor 2016* and fabricated using a commercial 3D direct laser writing setup [Photonic Professional GT, Nanoscribe GmbH, Stutensee, Germany (Niesler & Hermatschweiler, 2014 ▸; Maruo *et al.*, 1997 ▸)]. Two insert designs, with either a cone opening tip or a straight opening tip, were used (Figs. 2 ▸ and S2). The exterior of the cone opening design is shown as a CAD-rendering in Fig. 2 ▸(*a*). The tip of the insert in this design has a cone shape with a 250 µm diameter and contains a cross-shaped homogenizer so that the mixed flow emerges at its fully expanded diameter [Fig. 2 ▸(*b*)]. This design is ideal for capturing fast reaction time points, *e.g.* immediately after mixing, which requires data aquisition a short distance from the tip. Fig. S2(*a*) shows the exterior of the second, straight opening design, which has a tip that is 150 µm in diameter [Fig. S2(*b*)]. When the mixed sample exits this insert, it needs to expand to its final width of 250 µm. More information about the second design can be found in the supporting information.

For both designs, fins center the insert in the observation tube while allowing the sheath to flow around it, as shown in the orthographic view in Fig. 2 ▸(*a*). The supply line ports each accommodate a 200 µm outer diameter fused silica capillary (Polymicro Technologies, Phoenix, AZ), which can be glued in place with UV curable ep­oxy (Master Bond Inc. Hackensack, NJ). Constricted regions center the supply lines in the ports. For the cone opening design, fluids leaving each supply line are combined in a ∼1 mm-long, 100 µm-diameter channel containing the Kenics mixing elements and then exit through the cone-shaped ending [Fig. 2 ▸(*e*)]. Eight helical mixing elements are present in the cone opening design. Given our simulation results, 8 elements in a 100 µm channel produce ∼200 nm-thick layers of fluid. For a typical protein [diffusion coefficient ∼10^−11^ m^2^ s^−1^ (Young *et al.*, 1980 ▸)], near-complete mixing is achieved within several milliseconds. The insert can be modified for different mixing efficiencies if desired.

#### 3.1.3. Mixing insert and sample cell fabrication

Mixing inserts were 3D printed and completed inserts were stored in the developer until needed. Inserts were rinsed with iso­propanol to remove developer and allowed to dry fully before assembly (Knoška *et al.*, 2020 ▸). More details of device fabrication are provided in the supporting information and Figs. S3–S4. In brief, the process entails bonding two supply lines to the mixer, surface treating the mixer to make it less hydro­phobic (Sobiesierski *et al.*, 2015 ▸; Wang *et al.*, 2005 ▸), and then using customized sample cell holders to place the mixer inside thin-walled X-ray compatible glass tubing (Hilgenberg GmbH, Malsfeld, Germany) to complete the device.

#### 3.1.4. Mixing times and computing final time point probed

**Figure 3:**
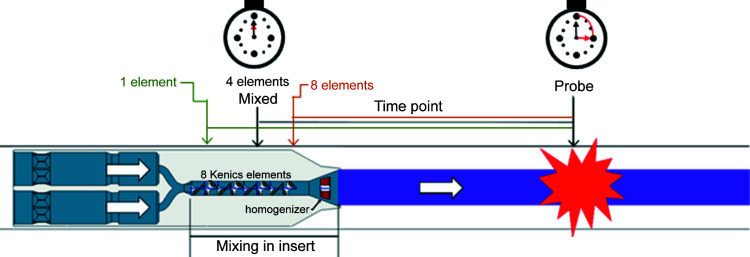
Graphical depiction of mixing and the time point probed. The different mixed points are indicated. Note that the full 8 elements are always present, but the final mixing point occurs at different places depending on the systems probed.

Mixing is considered complete when the concentration of the reacting species is 1:1 in the mixer; every molecule theor­etically has access to a binding partner. In both designs, the mixing occurs as the two solutions traverse the insert, but the point at which full mixing is achieved depends on the size and diffusion coefficient of the molecules being mixed and the viscosity and densities of the buffers. For example, mixing a macromolecule (protein or nucleic acid) with a small additive (ligand or ion) will be faster than mixing of two large macromolecules (two proteins or a protein and a nucleic acid). Therefore, we consider the relative size of the species being mixed to determine how many elements are required to fully initiate the reaction. This mixing time can be several milliseconds or longer, depending on the flow rates. We use the following guiding principles to account for these differences: two large species are considered fully mixed after 8 helical elements, one large species and one intermediate ligand are fully mixed after 4 helical elements, and one large species and one small species are fully mixed after 1 helical element (Table 1 ▸). Of course, the molecules pass through all 8 elements, even if they are fully mixed after 4. This 8-element design provides maximum flexibility for the different types of reactions that can be probed, without hindering the ability to probe even the fastest time points (∼10 ms) for fast reactions. The total time spent in the mixer can be kept short in most cases, but to account for some molecules reacting before all molecules in the sample are fully mixed, we approximate the uncertainty due to the travel time as half of the average transit time. Because most particles transit the mixer in about the same amount of time, this design presents a distinct advantage for time-resolved studies where differences in travel time through the device (residence or transit time) can dramatically increase error bars on mixing times or time points (Galaktionov *et al.*, 2003 ▸). A schematic of the different mixing initiation points is shown in Fig. 3 ▸.

Once the fully mixed point has been determined, the flow rates and the distance from the tip of the insert dictate the final time point probed (schematic in Fig. 3 ▸), based on an analytical solution of the Navier–Stokes and continuity equations. The details of the solutions and application of boundary conditions are reported in the supporting information. Briefly, a solution can be determined for flow velocities (*u*) of both sheath (*u*_sh_) and sample (*u*_s_) as a function of the radial direction of the sample cell (*r*, where *R* is the radius of the sample cell and *r*_s_ is the radius of the inner sample stream), the pressure gradient along the *z* axis (*G* = −d*P*/d*z*), the acceleration due to gravity (*g*), the viscosity of the fluids (μ_sh_, μ_s_) and the density of the fluids (ρ_sh_, ρ_s_).

For this work, all samples studied were water soluble and contained no additives that changed their viscosities or densities appreciably; thus we examine the case of 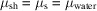 and 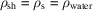 . In this regime, the simplified solutions are

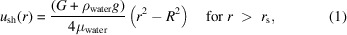




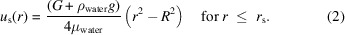

In addition to the time elapsed in the insert, uncertainties in the time point arise from the transit time of the sample through the area illuminated by the X-ray beam and from the parabolic flow profile of the sample traveling through the observation tube. These must be combined with the mixing time to find the total uncertainty in a measured time point. More details about these calculations and converting flow velocity to volumetric flow rate can be found in the supporting information.

For X-ray scattering experiments, a 250 µm diameter sample stream (surrounded by a sheath) represents a good balance between sample consumption, time point dispersion and path length (thickness of the sample that is illuminated by the beam). Given the diameter of the outlet channel (550 µm), the chosen diameter of the sample stream determines the ratio of sample to sheath flow. With this ratio clearly defined, absolute flow speeds determine the accessible time regime. Access to shorter time points requires fast mixing and fast flow, both of which occur at higher sample flow rates, while access to longer time points can tolerate slower mixing and slower flow, with lower sample flow rates. With either Kenics design, timescales ranging from milliseconds to seconds can be easily accessed by varying the flow speeds of both components of the sample, and the sheath, and each time point can be reached by multiple combinations of flow speeds and distances from the tip. However, it is important to note that the flow speed affects mixing times and therefore the overall uncertainty associated with each time point. Selection of experimental conditions is a compromise between lower uncertainty (higher sample flow rate) and less sample consumption (lower flow rate).

The accessible time points span three orders of magnitude (∼10–1000 ms) and can be changed by varying the flow conditions and position of the X-ray beam relative to the end of the insert. Table 2 ▸ provides a summary of the time points, uncertainties and suggested X-ray locations attainable with each flow condition for reaction class 1 (two large macromolecules), using a cone opening device with a 250 µm X-ray sample path length in a 550 µm inner diameter sample cell. It also shows how different flow conditions can be used to reach the same time point, so that sample consumption and timing uncertainty can be balanced based on the needs and limitations for a specific system. A discussion of uncertainty calculations is provided in the supporting information. Additionally, the flow conditions for the other reaction classes and for the straight opening device (Tables S1–S4) and the reproducibility of the same time points with different flow conditions are also included in Fig. S5.

#### 3.1.5. Mixer characterization using visible absorbance

**Figure 4:**
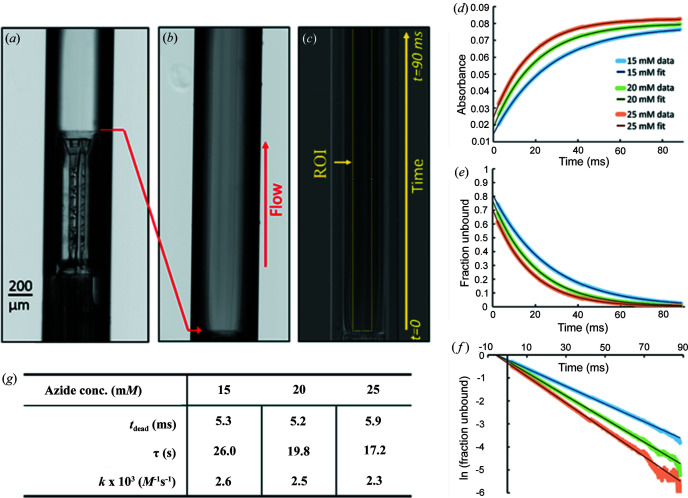
Myoglobin and azide time-resolved absorbance measurements in the cone opening device. Images of the cone opening Kenics and observation region during the absorbance experiment. (*a*) Cone opening Kenics in the observation tube. (*b*) Dark-subtracted image of the observation region for myoglobin mixing with azide. Striations visible in the image result from the cross-shaped homogenizer and occur in the reference image as well. (*c*) Image showing transmission. (*d*) Absorbance versus time for each of the three azide concentrations, with fits overlaid. The legend applies to all panels. (*e*) Fraction of unbound myoglobin versus time. (*f*) Log plot of fraction unbound versus time. The slight discontinuity in the data at ∼45 ms can be attributed to a mismatch in the two modules of the camera detector. (*g*) Table showing the dead time (*t*_dead_), time constant (τ) and reaction constant (*k*) for each azide concentration. Notably, the *k* values are a good match to the literature (2.8 ± 0.3 × 10^3^ *M* ^−1^s^−1^; Nami *et al.*, 2016 ▸). These values were extracted from fitting the absorbance versus time data.

Before mixers were used for TR-SAXS, control experiments were performed to assess their operation and efficacy. When azide binds to metmyoglobin, a change in absorbance occurs at several wavelengths in the UV–vis spectrum, including from about 550 to 575 nm (Marcoline & Elgren, 1998 ▸). This absorbance change can be easily captured in a custom-built long working distance microscope [described previously by Calvey *et al.* (2016 ▸)] so visible absorbance data on the myoglobin and azide system were acquired in a time-resolved experiment to characterize the effectiveness of both mixers, measure reaction kinetics and determine the dead time of the mixers [Figs. 4 ▸(*a*)–4(*c*)].

Fig. 4 ▸(*d*) shows absorbance versus time for the reaction of myoglobin with different concentrations of azide in the cone opening Kenics. The data are well fit with a single exponential, as expected for the pseudo-first order chemical reaction, with the azide in vast excess:

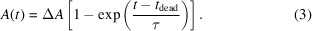

Here Δ*A* is the total change in absorbance, *t*_dead_ is the dead time (time between the beginning of the reaction and the point at which observations begin) and τ is the time constant of the reaction. Note that the dead time is context-dependent; it varies according to the diffusion coefficients of the species being mixed, as well as the flow rates. The rate constant *k* can be determined according to

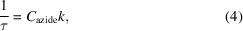

where *C*_azide_ is the azide concentration after mixing.

The absorbance data can be converted to report the fraction of unbound myoglobin over time [Fig. 4 ▸(*e*)]. As expected, higher azide concentrations result in faster decay in the unbound myoglobin fraction. In Fig. 4 ▸(*f*), the log of the fraction of unbound myoglobin is plotted. As expected from the pseudo first-order reaction, the data are well fit with a straight line. Here, the dead time is the time intercept and −1/τ is the slope.

The kinetics properties extracted from the fits are shown in Fig. 4 ▸. The measured *k* values from this experiment agree well with the literature value 2.8 ± 0.3 × 10^3^ *M* ^−1^s^−1^ for the same buffer conditions (Nami *et al.*, 2016 ▸), demonstrating that the insert mixes properly and that this device can be used to accurately measure reaction kinetics. Results from the straight-opening design and the derivation of the formulae used to determine the rate constant and dead time are shown in the supporting information. Even with modest combined sample flow rates of 110 µl min^−1^, the dead time is low enough to probe reaction time points below 10 ms, sufficient to capture side-chain motions and allosteric transitions (Benkovic & Hammes-Schiffer, 2003 ▸). Higher flow rates could be used to access shorter dead times in this device, if desired. Overall, these absorbance measurements show that this device is a robust tool for measuring chemical kinetics if proper sample preparation and filtering steps are taken.

### 3.2. Mixer applications to TR-SAXS

To demonstrate the effectiveness of the Kenics mixer and its compatibility with SAXS, we used the different mixer designs to examine a broad range of reacting species. Reactions between molecules of different sizes were recorded, and different time ranges were accessed using the two different insert geometries. In this section, we report results on a variety of systems that highlight the versatility and breadth of the design. SASBDB accession codes for all data are provided in the supporting information.

#### 3.2.1. Comparing diffusive and chaotic advection mixing for magnesium-driven GAC rRNA folding

**Figure 5:**
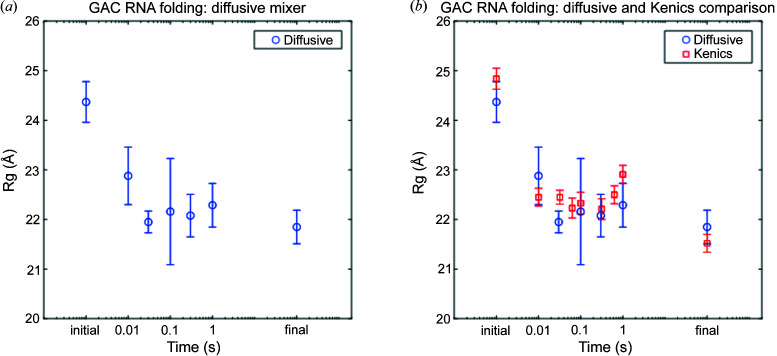
GAC rRNA folding experiment. (*a*) The mean *R*_g_ of the RNA at different time points as the RNA folds and becomes more compact, as collected previously with the diffusive mixer (Welty *et al.*, 2018 ▸). (*b*) Comparing the folding of the RNA by looking at the *R*_g_ as a function of time with the diffusive mixer (blue) and Kenics device (red). The error bars are smaller for the Kenics data points because the reaction initiation is tighter so there is less of a spread in the age of the population.

The first experiment examined Mg^2+^-initiated RNA folding. RNA in buffered solutions containing monovalent salt possesses secondary but not tertiary structures. For many RNA molecules, Mg^2+^ facilitates the formation of stabilizing, tertiary contacts (Draper, 2004 ▸). The system of interest is a 60-nucleotide GTPase center (GAC) ribosomal RNA (rRNA), whose folding was previously studied using a diffusive mixer (Welty *et al.*, 2018 ▸). For these past experiments, GAC rRNA, in a monovalent salt solution, flowed through a central channel and Mg^2+^ was introduced via a coaxially flowing solution. The GAC rRNA was flow-focused into a thin stream to facilitate rapid mixing. We collected an initial state (no Mg^2+^), steady state (GAC rRNA incubated with Mg^2+^ for several minutes) and five intermediate time points (10, 30, 50, 100, 300 and 1000 ms) to track the progression of this RNA folding reaction [Fig. 5 ▸(*a*)]. Although structural changes were recorded over a broad range of scattering angles, as described in our previous publication, for the purposes of this study we focus on a single parameter, the overall size of RNA reported by its radius of gyration, *R*_g_. Before the addition of Mg^2+^ to trigger folding, the *R*_g_ of the RNA was 24.37 ± 0.41 Å. Within 10 ms of the addition of Mg ions, the *R*_g_ decreases to 22.88 ±0.58 Å. Small changes in the *R*_g_ are observed as the reaction is followed to 1000 ms. By this time, *R*_g_ = 22.29 ± 0.44 Å, which agrees within error with the steady state value of 21.85 ± 0.34 Å.

We repeated this experiment with the Kenics mixer (cone opening device, reaction class 3) with GAC rRNA and Mg^2+^ flowing through separate channels until they were rapidly combined inside the Kenics insert. We collected the initial, Mg^2+^-free state, a steady state point (GAC rRNA incubated with Mg^2+^) and seven intermediate time points (10, 32, 63, 100, 316, 631 and 1000 ms) to track reaction progress [Fig. 5 ▸(*b*)]. Good agreement is found when comparing the *R*_g_ determined by the Kenics and diffusive mixers. In both cases, we observe a large, rapid compaction on the ∼10 ms timescale, which results in a transient state with comparable *R*_g_. The ensuing fluctuations in *R*_g_ through reaction times that reach 1000 ms are virtually identical between the two mixers. The *R*_g_ measured at 1000 ms in the Kenics device is 22.91 ± 0.18 Å, which is close to the *R*_g_ measured in the diffusive mixer at 1000 ms (22.29 ± 0.44 Å). This agreement demonstrates that the Kenics mixer accurately recapitulates prior TR-SAXS results for reactions of large and small species. Most notable is the reduction in the uncertainty of *R*_g_ in the Kenics mixers relative to its value obtained with the diffusive mixer. This sharpening likely results from the more efficient reaction initiation in the Kenics, and the reduced spread in reaction age, although other beamline and data collection improvements may also contribute. As shown in Fig. 1 ▸, the baker’s transformations, which initiate mixing inside the Kenics, create thin layers of liquid (200 nm), so the reaction initiation is almost uniform, especially for mixing with small, rapidly diffusing species such as Mg^2+^. This rapid and complete mixing stands in contrast with flow-focused mixing. In the former case, diffusion proceeds rapidly over a very short distance of ∼200 nm, while the central focused jet of the diffusive mixer can be a few micrometres in diameter. Key to rapid, uniform mixing is a minimization of the length scale for diffusion, which is only achieved by the Kenics.

#### 3.2.2. Capturing transient states of the Ca^2+^-driven self-association of tissue-transglutaminase

A second application of the Kenics mixer that leverages the need to rapidly reach and maintain a fixed, final ligand concentration, is the study of some protein conformational changes. In the Kenics, mixing of the two species occurs quickly and uniformly. In contrast, in a diffusive mixer, the concentration of the small (diffusing) ligand steadily increases over time, which suffices for reactions thst require a concentration threshold, but is not ideal for systems that are sensitive to the total ligand concentration. One system that benefits from the Kenics scheme is tissue-transglutaminase (tG) in the presence of Ca^2+^. tG is an enzyme that has distinct ‘open’ and ‘closed’ states, which are modulated by the presence of Ca^2+^ and GTP. Ca^2+^ drives tG open and in this state, tG acts as a crosslinker. GTP closes tG, and in this state, tG acts as a GTPase (Liu *et al.*, 2002 ▸; Pinkas *et al.*, 2007 ▸). The increased expression of tG is linked to several different cancers (Mann *et al.*, 2006 ▸; Katt *et al.*, 2022 ▸). Interestingly, when tG is mutated to stay in an open conformation, it is toxic to cells (Datta *et al.*, 2007 ▸; Katt *et al.*, 2018 ▸) for reasons that are poorly understood. Thus, there is significant interest in studying this conformational change and exploring ways to stabilize the open state for therapeutic purposes.

**Figure 6:**
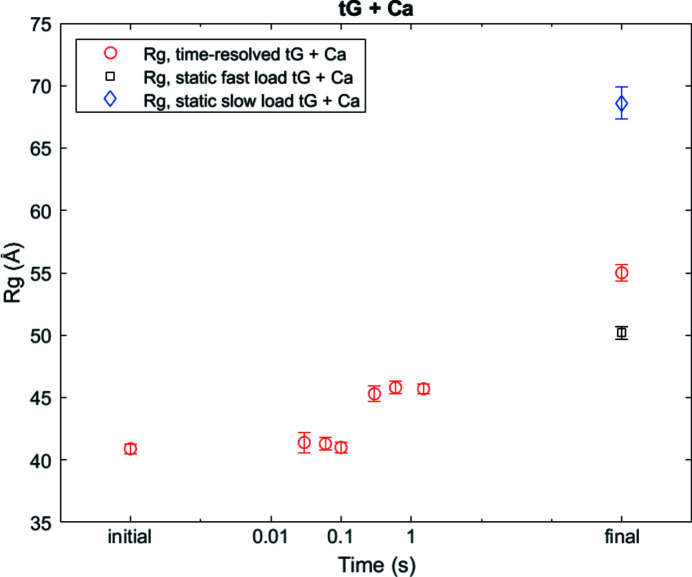
The *R*_g_ of tG as calcium binds and triggers a conformational change. Three different endpoints are also shown to demonstrate the aggregation of tG at longer time points that are typical for conventional equilibrium SAXS measurements. Fast load corresponds to a 5 min incubation and slow load corresponds to a 30 min incubation.

We first studied the tG + Ca^2+^ reaction with static SAXS and found that the opening process was sensitive to both the concentration of Ca^2+^ and the reaction incubation time (Fig. 6 ▸). When tG was incubated with Ca^2+^ for 5 min (fast-load), the final *R*_g_ was 50.2 ± 0.5 Å, but when incubated for 30 min (slow-load), the *R*_g_ increased to 68.6 ± 1.3 Å. This longer incubation time with Ca^2+^ results in more aggregation, as indicated by the increase in the SAXS intensity at zero angle, and the amount of aggregation seems to correlate with Ca^2+^ interaction time.

Due to the formation of higher order oligomers on the minute timescale, equilibrium SAXS cannot capture the first stages in the opening of tG. In contrast, TR-SAXS offers the opportunity to capture this transient state with the potential to capture the monomeric form and elucidate the mechanism of tG opening. We used the Kenics mixer [straight opening device (Fig. S2), reaction class 3] to study this process. We captured an initial state (no Ca^2+^), a steady state (tG incubated with Ca^2+^ for approximately 10 min) and six intermediate time points (32, 63, 100, 316, 631 and 1500 ms; Fig. 6 ▸).

We were surprised to find two relatively stable states with a sharp transition between 100 and 316 ms. We captured a transition from the initial state of tG, which is believed to be a mixed state of open and the dimer form of tG, and observed that the overall dimer fraction increases (manuscript in preparation). This transition was undetectable with static SAXS, and the Kenics mixer was the ideal tool to capture the monomer-to-dimer transition of tG, especially since our static SAXS measurements show the formation of aggregates on timescales of 5–30 min.

#### 3.2.3. Binding of trypsin and aprotinin

**Figure 7:**
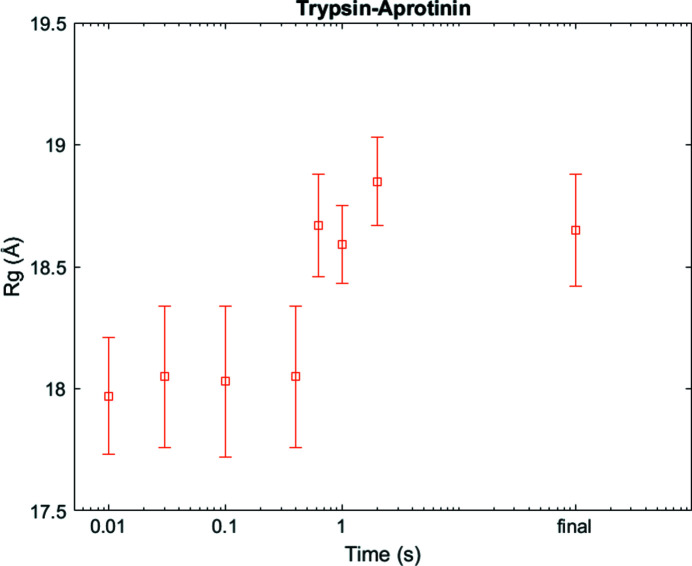
Changes in *R*_g_ as trypsin and aprotinin bind, even these small changes can be captured.

The above examples illustrate the mixing of large and small reacting species. However, the most unique feature of the Kenics insert is its ability to rapidly combine and follow reactions between two large species. Reactions between two macromolecules can also be studied with a stopped-flow mixer, but the Kenics approach reduces sample consumption. As proof of principle, we examined binding of a model system comprised of the proteins aprotinin and trypsin. We flowed trypsin through one side of the Kenics (cone opening device, reaction class 1) and aprotinin through the other so that we could observe the time evolution of the reaction. We collected the two initial states (trypsin alone and aprotinin alone), a steady state (trypsin and aprotinin incubated together) and seven intermediate time points (10, 32, 100, 400, 631, 1000 and 2000 ms; Fig. 7 ▸). Again, using the *R*_g_ as the reaction metric, we measured a steady increase with time, indicating binding. At 2000 ms, the complex appears to be already fully formed. Because aprotinin is a small protein, with a molecular weight of 7 kDa, its binding to trypsin (24 kDa) does not yield a large change in the overall *R*_g_. However, it is too large (diffusion too slow) to mix via a flow focusing diffusive mixer. We also used this model system to assess the reproducibility of the Kenics mixer. Importantly, we showed that the same time point could be measured with different flow conditions, by adjusting the measurement position distance from the tip, to yield nearly identical scattering profiles (Fig. S5).

#### 3.2.4. Evolution of GAC rRNA + L11 protein complex

**Figure 8:**
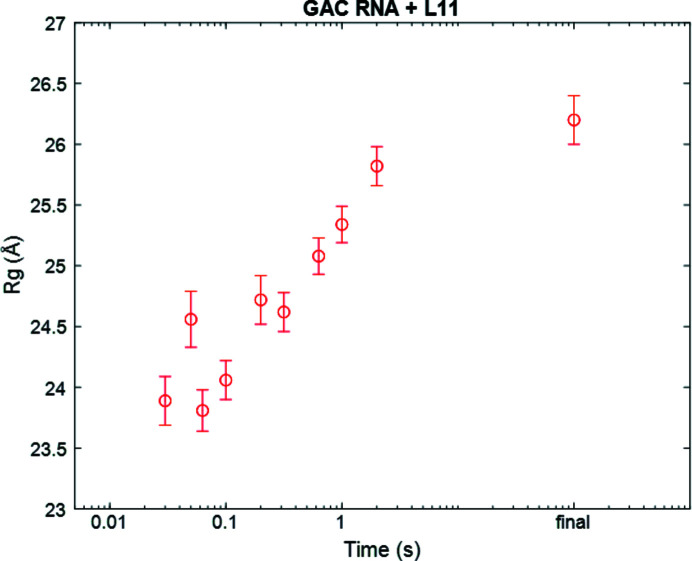
Change in *R*_g_ on the association of GAC rRNA with its L11 protein partner.

This last example considers mixing of two comparably sized macromolecules, an rRNA fragment with a molecular weight of 18.7 kDa and a protein domain with a molecular weight of 15.6 kDa. Protein–nucleic acid complexes, especially RNA–protein complexes, are biologically important, yet poorly understood. The chaotic advection mixer provides the opportunity to study the structural changes that immediately follow (or precede) the binding of two moderately sized macromolecules. The small GAC rRNA discussed above is part of the 23S bacterial ribosome. In the ribosome, its biological partner is the protein L11. Crystal structures show that the protein stabilizes the tertiary fold of the functional RNA (Blyn *et al.*, 2000 ▸). In previous work (Welty *et al.*, 2020 ▸), we stipulated that the C-terminal domain of the L11 protein binds RNA during its Mg^2+^-dependent folding trajectory. Here, we examine binding of the RNA to the full length L11 protein (Jonker *et al.*, 2007 ▸). To study this reaction, we used the Kenics (cone opening device, reaction class 1) to mix unstructured GAC rRNA in KCl with L11 protein in MgCl_2_. We collected two initial states (GAC rRNA alone, no Mg^2+^ and L11 alone, with Mg^2+^), one final state (GAC rRNA, L11 and Mg^2+^ incubated together for several minutes) and ten intermediate time points (30, 50, 63, 100, 200, 316, 631, 1000 and 2000 ms; Fig. 8 ▸). As we mixed GAC rRNA with L11 in MgCl_2_, the *R*_g_ steadily increased with time, supporting the model that L11 stabilizes the folded RNA as they bind.

## 4. Discussion

Here we demonstrated that a chaotic advection mixer, with a Kenics design, is extremely versatile and enables triggering and monitoring of reactions involving widely sized pairs of reactants. Its use was demonstrated in four different experiments, following the mixing of RNA with ions (GAC rRNA with Mg^2+^), protein with ions (tG with Ca^2+^), protein with protein (trypsin with aprotinin) and RNA with protein (GAC rRNA with L11 protein). In each case, we observed dynamics on timescales relevant for conformation dynamics in many systems, ranging from 10 ms through 2000 ms. The high-efficiency mixing of the Kenics design, based on a baker’s transformation, is the key to its flexibility when applied to this broad ranging group of reactions. The resulting fast mixing also leads to lower uncertainty in each measurement, illustrated by the smaller size in the error bars for the *R*_g_ measurements carried out with a Kenics mixer and a flow-focusing counterpart [Fig. 5 ▸(*b*)]. This lower uncertainty is a particular advantage when measuring at the fastest time points, and especially for processes that can complete on this timescale. The decreased spread in overall age of the reaction means that distinct intermediates will be easier to resolve.

We also described an efficient and sample-minimizing loop-loading system that requires 75 µl of sample and 150 µl of buffer for each time point. This volume is on par with what is required for a single SAXS measurement at most synchrotrons. It represents a significant reduction from the approximate hundreds of microlitres per time point required for both turbulent and stopped-flow mixers. Additionally, the continuous-flow nature of Kenics mixer still allows for radiation damage limits to be avoided, like the turbulent mixer, but at much lower flow rates, due in part to the sheath flow surrounding the sample stream. With this moderate sample consumption, an entire time series with 7 time points can be collected with only ∼500 µl of sample and in approximately 3 h (including cleaning times and reloading), allowing for collection of multiple time series with different molecules during a single, days-long beam time. This efficiency is particularly advantageous when performing mixing experiments with the same biomolecule, but different ligands, as a single batch of the biomolecule could be used. Thus, experiments probing the interaction of a single protein with different partner molecules can be performed under as close-to-identical conditions as possible to help answer biological questions.

Furthermore, there is a large demand for structural information of ligand binding events to help drive drug design and even the moderate resolution range of SAXS is sufficient to gain some mechanistic understanding of these events (Aplin *et al.*, 2022 ▸). Both our tG + Ca^2+^ and trypsin + aprotinin experiments demonstrate that protein dynamics and structural information can be captured with our device. Additionally, our myoglobin and azide time-resolved absorbance experiments show that accurate *k* values can be extracted from data taken with our Kenics mixer. Therefore, this mixer may contribute to time-resolved structural studies of drug–target interactions through both structural and kinetic measurements. Because accurate determinations of kinetic rate parameters require measurements at known, fixed concentrations of small compounds, the Kenics style mixer is very well suited for these measurements. There is the potential to use the structural mechanistic information gained by TR-SAXS and kinetics experiments with the Kenics to alter ligand designs to go from micromolar to nanomolar binding affinities.

Finally, because of its flexible design, the mixing insert can be combined with additional structural or spectroscopic probes that provide information complementary to SAXS. These mixers can be readily coupled with simple-to-design outlet configurations that enable measurements by X-ray spectroscopies, crystallography or cryo-EM techniques. In addition, due to the ease of fabrication by 3D-printing, mixing can be optimized for systems containing larger particles such as microcrystals. The versatile mixer system described here can be coupled with a wide variety of mix-and-inject systems. With the sample efficient loading described herein, a broad range of biologically important reactions can be probed on the timescales relevant to conformational dynamics.

## 5. Conclusions

We report the design, operation and use of a 3D-printed Kenics-style chaotic advection mixer to probe reactions involving classes of biological macromolecules with each other, or with a wide variety of small molecules. The information revealed will improve our understanding of important biophysical and medical processes, ranging from probing the underlying physics of macromolecular self-assembly to improving structure-based drug design.

## 6. Related literature

The following reference is cited in the supporting information: Squires & Quake (2005 ▸).

## Supplementary Material

SASBDB reference: SASDRE3

SASBDB reference: SASDRF3

SASBDB reference: SASDRG3

SASBDB reference: SASDRH3

SASBDB reference: SASDRJ3

SASBDB reference: SASDRK3

SASBDB reference: SASDRL3

SASBDB reference: SASDRM3

SASBDB reference: SASDRN3

SASBDB reference: SASDRP3

SASBDB reference: SASDRQ3

SASBDB reference: SASDRR3

SASBDB reference: SASDRS3

SASBDB reference: SASDRT3

SASBDB reference: SASDRU3

SASBDB reference: SASDRV3

SASBDB reference: SASDRW3

SASBDB reference: SASDRX3

SASBDB reference: SASDRY3

SASBDB reference: SASDRZ3

SASBDB reference: SASDR24

SASBDB reference: SASDR34

SASBDB reference: SASDR44

SASBDB reference: SASDR54

SASBDB reference: SASDR64

SASBDB reference: SASDR74

SASBDB reference: SASDR84

SASBDB reference: SASDR94

SASBDB reference: SASDRA4

SASBDB reference: SASDRB4

SASBDB reference: SASDRC4

SASBDB reference: SASDRD4

SASBDB reference: SASDRE4

SASBDB reference: SASDRF4

SASBDB reference: SASDRG4

SASBDB reference: SASDRH4

SASBDB reference: SASDRJ4

SASBDB reference: SASDRK4

SASBDB reference: SASDRL4

SASBDB reference: SASDRM4

SASBDB reference: SASDRN4

SASBDB reference: SASDRP4

SASBDB reference: SASDRQ4

SASBDB reference: SASDRR4

Supporting text, tables and figures. DOI: 10.1107/S2052252523003482/ro5036sup1.pdf

## Figures and Tables

**Table 1:** Three reaction classes, based on the relative size of the species mixed

Reaction classes	Species A	Species B	Number of mixing elements needed for full mixing
Two large species	Protein	Protein	8 elements (full insert)
	Protein	DNA	8 elements (full insert)
	Protein	RNA	8 elements (full insert)
1 large biomacromolecule + 1 intermediate-sized ligand	Protein	Ligand	4 elements (half insert)
	DNA	Ligand	4 elements (half insert)
	RNA	Ligand	4 elements (half insert)
1 large biomacromolecule + 1 small-sized ion	Protein	Ion	1 element (eighth of insert)
	DNA	Ion	1 element (eighth of insert)
	RNA	Ion	1 element (eighth of insert)

**Table 2:** Flow conditions for reaction class 1: two large biomacromolecules in the cone opening device

Sample A flow rate (µl min^−1^)	Sample B flow rate (µl min^−1^)	Sheath flow rate (µl min^−1^)	Time point (ms ± uncertainty)	Distance from tip (µm)
60	60	203.1	10 ± 6	293
60	60	203.1	20 ± 6	705
30	30	101.5	20 ± 12	292
30	30	101.5	50 ± 12	917
30	30	101.5	100 ± 13	1952
20	20	67.7	100 ± 18	1262
20	20	67.7	250 ± 23	3354
10	10	33.8	250 ± 38	1615
10	10	33.8	500 ± 46	3344
10	10	33.8	1000 ± 71	6793
