# Supplementary material for: Chaotic advection mixer for capturing transient states of diverse biological macromolecular systems with time-resolved small-angle X-ray scattering
Source: IUCrJ. 2023 Apr 28;10(Pt 3):363–75. doi: 10.1107/S2052252523003482 (PMC10161774; doi:10.1107/S2052252523003482)
Supplement: Supplementary file 1 [file m-10-00363-sup1.pdf]

IUCrJ

Volume 10 (2023)

Supporting information for article:

Chaotic advection mixer for capturing transient states of diverse biological macromolecular systems with time-resolved small angle X-ray scattering

Kara A. Zielinski, Andrea M. Katz, George D. Calvey, Suzette A. Pabit, Shawn K. Milano, Cody Aplin, Josue San Emeterio, Richard A. Cerione and Lois Pollack

S1. Methods

S1.1. SAXS Experiments: Sample Delivery Setup

This complex setup (Figure S1) has three main sub-systems: the oil pumps and refill system (on the pump tower, A.i. and B.i.), the sample and buffer injection loops (on the valve shelf, A.ii and B.ii), and the loop cleaning station (B.iii.).

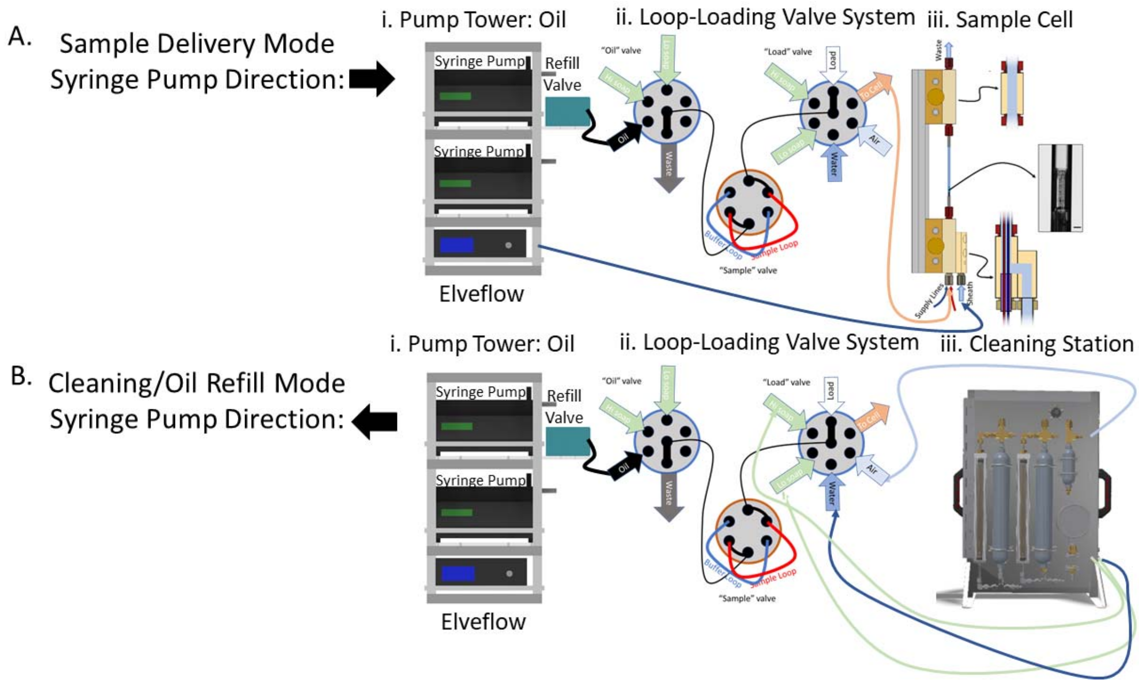

Figure S1 Schematic of the loop-loading sample delivery system. A. Shows the system in sample delivery mode. The syringe pumps (i.) are switched on in "infuse" mode and push mineral oil through the valves and loops (ii.) to bring the sample and buffer to the mixer for data collection (iii.). Arrows show the direction of flow. B. Shows the system is cleaning and oil refill mode. The syringe pumps (i.) are switched on, but in "refill" mode. The oil refill valve is set to the refill position and the Elveflow pressurizes the oil reservoirs to drive the oil into the syringes. The valves (ii.) are set to receive soap, water, and nitrogen gas from the cleaning station (iii.). Each species is delivered sequentially to clean the buffer and sample loops so that fresh samples can be loaded. Arrows show the direction of flow.

For data collection, the sheath flow is always on and is comprised of either the buffer alone for Reaction Class 1 (two large macromolecules) or the buffer with the ligand or ion for Reaction Classes 2 and 3 (one large macromolecule with a ligand or one large macromolecule with an ion, respectively). The following protocol is used to control the valves and loop-loading sample delivery system and demonstrated by the arrows in Figure S1. We have developed software to control all these processes remotely from outside the hutch, so that data can be collected uninterrupted. It takes about

10 minutes to collect a buffer, sample, buffer series for each timepoint (Steps 1-10). It then takes an additional 10 minutes to run the clean and refill cycle (Step 11).

1. Valves are switched to the ‘load’ position.
2. Syringes are connected to the load valve with a needle port and both the buffer and sample loops for each side of the mixer are filled.
3. Valves are switched to the ‘run’ position for the buffer, and the syringe pumps are turned on to start pushing oil to drive the buffers into the mixer. The flowrate of the oil and the total flow rate through the mixer are monitored with flow meters.
4. Once the total flowrate has reached the target value, data collection begins for the ‘pre-buffer’.
5. Data collection should stop when the buffer loop is half empty, and at this point, the syringe pumps are switched off.
6. Valves are switched to the ‘run’ position for the sample, and the syringe pumps are turned back on. The flowrate of the oil and the total flow rate through the mixer are again monitored with flow meters. Additionally, a minimum purge volume to clear all the buffer out of the lines is flown through.
7. Once the total flowrate has reached the target value and the purge volume has flowed, the sample data collection can begin.
8. Data collection should stop when there are approximately 5-7 microliters left in the loop to prevent oil from ever entering the sample cell, and at this point, the syringe pumps are switched off.
9. The valves are switched back to the ‘run’ position for the buffer, and the syringe pumps are turned back on. The flowrates and purge volume are both monitored before starting data collection for the ‘post buffer.’
10. Data collection should stop when there are approximately 5-7 microliters left in the loop to prevent oil from entering the sample cell. Now, one datapoint has been fully collected.
11. The loops are then cleaned with soap and water and dried with nitrogen gas so that they are ready for the next sample. At the same time, the oil syringes are refilled through an automated process.

There are many benefits to this loop loading setup, namely that high quality data can be achieved while still conserving samples and performing experiments in a timely manner. To ensure good data quality, this scheme allows for accurate buffer subtraction by producing a repeatable path length for sample and buffer scans. The sample and buffer species are driven by the same syringe pump, so the same conditions are reached for both sample and buffer scans, which is key for a good match.

Additionally, using an immiscible oil to drive the species out of the loops prevents sample dilution, therefore a constant concentration of sample is maintained throughout the measurement. To reduce sample consumption, the loops are dried with nitrogen gas as part of the cleaning cycle, which removes the need to flush the loops when loading and further prevents sample dilution. Also, the loops are positioned as close to the sample cell as possible, which cuts down on dead volume. The loops are easy to load, and it is recommended that several (3-5) excess μL of sample and buffer are loaded to ensure that any bubbles introduced while making the needle port connection are flushed backwards through the loop and out into the waste. To guarantee speedy data acquisition, the system was designed to reach stable, repeatable flowrates as efficiently as possible. Specifically, the overall volume of the oil syringes is kept low (100-250 μL), which allows for target flowrates to be reached quickly. With the automatic refill system, the syringes are simply refilled between datasets. Also, all downstream fluidic components have small inner diameters and there are no O-rings or soft tubing that would cause major pressure fluctuations or impact the overall flow stability. All components were carefully considered to keep flow stability and efficiency as top priorities. Lastly, the loop-loading system is also a highly flexible platform. Static measurements can easily be accommodated with minimal setup changes, allowing for easy comparison between static and time-resolved data within the same beamtime and under the same conditions.

If desired, a manual approach, in which the samples and buffers are directly loaded into syringes, is also possible, but can be challenging. Stainless steel high-pressure syringes should be used to withstand the pressure caused by the higher flow rates and small inner diameter (75-100 micron) of the tubing connected to the sample cell. These syringes typically have a large volume (5-10 mL), so a lot of sample needs to be loaded at once, which is not ideal for purified proteins and some ligands that are available in limited quantities. These syringes can also be slow to pressurize when initiating the syringe pump. Additionally, when switching between samples or different conditions, the syringes need to be manually removed from the pumps, cleaned, and then refilled, which can be very time consuming when trying to collect multiple time series. Lastly, this system had a higher dead volume as the sample had to travel from the syringe, through the switching valve, and then finally to the mixer.

S2. Results

S2.1 Mixer Design

Figure S2 shows a second Kenics design, with a straight opening. The tip is 150 μm in diameter, so when the stream exits the insert, it still needs to expand to its full 250 μm diameter. The channel with the Kenics mixing elements is also 150 μm diameter (Figure S2E), which is slightly larger than the 100 μm diameter in the cone opening design. Additionally, there are only 5 elements in this mixer, as opposed to the 8 in the cone opening design. Although both the wider channel and fewer elements

result in slightly slower mixing, it can still be successfully used. The 5 elements in a 150 µm channel produce ~2000 nm thick layers.

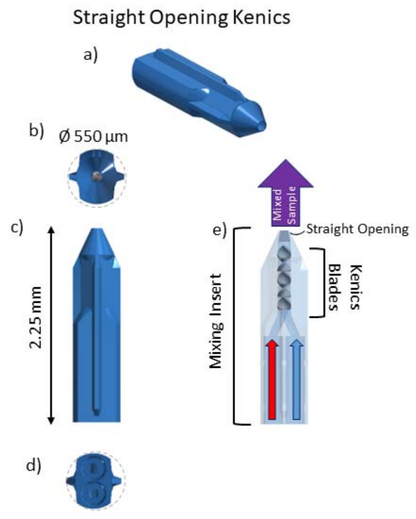

Figure S2 CAD rendering of the 3D printed mixing insert that houses the Kenics mixer. a) Exterior of the device. b) View of the straight opening from downstream. The dashed grey line represents the observation tube. c) Side view of the straight opening insert. d) View of the straight opening insert from upstream, showing the supply line ports. e) Cross-sectional view of the straight opening insert with the straight opening shaded in grey.

S2.2 Mixing Insert and Sample Cell Fabrication

S2.2.1 Supply Line Binding Details

Two, ~24” long capillary tubes with 75-100 µm inner diameter and 200 µm outer diameter (TSP100200, Polymicro Technologies, Phoenix, AZ) were inserted into the ports on the Kenics mixer with a three-axis translation stage. Next a sub microliter amount of UV curable epoxy (UV18S, Masterbond, Hackensack, NJ) is applied onto the gap between the two supply lines just behind the insert. When the epoxy bridged the two supply lines, the applicator was used to carefully draw it forward until it reached the back of the insert, where it could wick into the supply line ports. This application method was chosen because it gave a large amount of control and ensured the epoxy did not flow onto the outside of the insert or form a large blob that would have interfered with the passage of the sheath flow. When the epoxy had wicked to the bottom of the ports, it was rapidly cured from both sides with light from a 365 nm LED (LED Engin Inc., Marblehead, MA).

Figure S3A shows a picture of a mixing insert with supply lines bonded in. In subsequent mixer assembly steps, a seal must be made around these two supply lines using a single standard microfluidic port. To accomplish this, both supply lines were encapsulated by a larger glass tube, around which the seal can be made (Figure S3b). A 20 mm long piece of 550 µm ID, 794 µm OD

glass tubing (Drummond Scientific, Broomall, PA) was placed over the supply lines so that its closest end was 25 mm upstream of the mixing insert. Low viscosity UV curable epoxy (UV15, Master Bond) was wicked through the glass tube and cured.

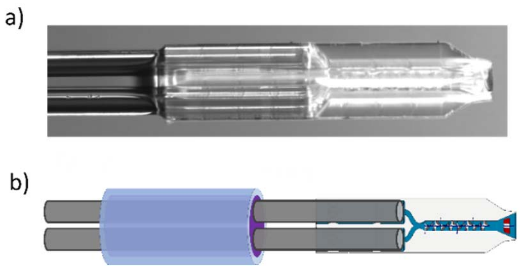

Figure S3 Insert with bonded supply lines. a) Photo of mixing insert after lines are bonded. b) Cartoon illustrating the bonding of the larger glass tube (light blue) over the supply lines to create a single seal. Epoxy is shown in purple. The large glass is positioned much further away from the insert than illustrated here.

S2.2.2 Surface Treatment for Reducing Insert Hydrophobicity

The epoxy-like material that the insert is made of is naturally hydrophobic. As a result, air bubbles inside of the insert are challenging to displace, and bubbles in the Kenics elements can interfere with mixing. Therefore, a surface treatment that renders the inserts less hydrophobic is beneficial. A modified version of a treatment often used for microfluidic devices made from SU-8 (Sobiesierski *et al.*, 2015; Wang *et al.*, 2005) works well for these inserts.

The surface treatment must be performed after the supply lines are glued into the insert. The first step is to use ceric ammonium nitrate (CAN) to catalyse a reaction in which nitric acid breaks open the epoxy rings on the surface of the insert. For the treatment to be effective, no bubbles can be present in the interior of the insert so that the chemical can access all of its interior surfaces. This was achieved by flowing water through each of the supply lines and the insert and then placing the insert in a sonicator for 10-20 s.

The insert was then quickly transferred to a beaker of 0.1 M ceric ammonium nitrate (CAN) in1 M nitric acid held at 50° C. During the transfer, water flow was kept on preventing air from re-entering the insert. Once the insert was submerged in the CAN mixture, the syringe pump was set to withdraw fluid through the insert at 5 μL/min on each line. This ensured that fresh CAN mixture was always circulating through the insert to react with the interior surfaces. This process was allowed to continue for 50 minutes. At this point, the color of the insert changes from yellow to green.

After the CAN treatment, the insert was thoroughly rinsed with distilled water to remove CAN from the supply lines and interior channels. This was crucial because the CAN mixture is insoluble in the chemical which forms the second stage of the treatment, so it must be thoroughly flushed out to avoid

clogging. The same sonication step was repeated to remove any bubbles that entered the insert during cleaning, and then the insert was transferred directly to a beaker of 0.1 M ethanolamine in 0.1 M sodium phosphate buffer at 50° C. The syringes were set to withdraw the ethanolamine mixture through the insert at 5 µL/min for 20 minutes. After this, the insert was removed and thoroughly flushed with distilled water. The green color remained. After surface treatment, bubbles were readily flushed out of the device. A high flow rate of water or soap (~100 µL/min) through the insert can remove any bubbles that do not dislodge during normal operation.

S2.2.3 Sample Cell Fabrication

Each mixer was assembled with custom components and mount, shown in Figure S4 a). A custom machined PEEK microfluidic tee (Figure S4 e) and union (Figure S4 c) were mounted on a rail for alignment as shown in Figure S4 b). Two short pieces of 1/32" OD, thin wall stainless steel tubing (89935K76, McMaster-Carr, Princeton, NJ) were secured to the downstream end of the tee and the upstream end of the union with standard 6-32 coned fittings (F-126, IDEX Health and Science, Oak Harbor, Washington). Then, a 2" long piece of 550 µm ID, 610 µm OD borosilicate glass (Code # 1472544, Hilgenberg GmbH, Malsfeld, Germany) to serve as the observation tube was suspended between the steel tubes and glued in place with five-minute epoxy. The insert assembly was threaded through the tee and into the observation capillary (Figure S4 d), then secured in place with another 6-32 fitting over the epoxy-filled glass tube to create a seal over both supply lines. A sheath supply line and waste line made from 1/32" OD, 380 µm ID PEEK tubing (TPK.515, Valco Instruments, Houston, Texas) were attached to finish the mixer assembly.

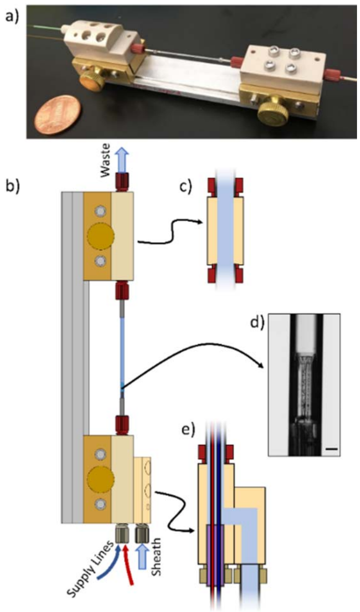

Figure S4 Assembled mixer. A) Picture of a completed device, with penny for scale. B) CAD image of the assembled device, with insets for detail: c) the union where the waste line is connected, d) the

mixing insert, and e) the tee that facilitates concentric flow of the sheath around the supply lines and insert.

S2.3 Mixing Times and Final Timepoint Probed

From the Navier-Stokes equations, the general solution for the flow velocity, u, along the channel before applying boundary conditions is:

$$u_{sh}(r) = \frac{r^2}{4\mu_{sh}}(G + \rho_{sh}g) + C_1 \ln(r) + C_2 \qquad \text{S1}$$

$$u_s(r) = \frac{r^2}{4\mu_s}(G + \rho_s g) + C_3 \ln(r) + C_4 \qquad \text{S2}$$

where u_{sh} and u_s are the flow velocities of the sheath and sample respectively, μ_{sh} and μ_s are the viscosities of the sheath and sample respectively, G is the pressure gradient along the z-axis (direction of flow; $-dP\ /\ dz$), ρ_{sh} and ρ_s are the densities of the sheath and sample respectively, g is the acceleration due to gravity (Squires & Quake, 2005). After applying boundary conditions, the four constants are:

$$C_1 = \frac{r_s^2}{2}\left(\frac{-1}{\mu_{sh}}(G + \rho_{sh}g) + \frac{1}{\mu_{sh}}(G + \rho_s g)\right) \qquad \text{S3}$$

$$C_2 = -C_1 \ln(R) - \frac{R^2}{4\mu_{sh}}(G + \rho_{sh}g)$$

$$C_3 = 0$$

$$C_4 = \frac{r_s^2}{4\mu_{sh}}(G + \rho_{sh}g) - \frac{r_s^2}{4\mu_s}(G + \rho_s g) + C_1 ln(r_s) + C_2$$

Where R is the radius of the sample cell and r_s is the radius of the inner sample stream.

The volumetric flowrate, $\dot{V}_{sh}$ and $\dot{V}_s$, for the sheath and sample is calculated by radially integrating the velocity to get:

$$\dot{V}_{sh} = 2\pi(\frac{R^4 - r_s^4}{16\mu_{sh}}(G + \rho_{sh}g) + C_1\left(\frac{R^2}{4}(2\ln R - 1) - \frac{{r_s}^2}{4}(2\ln r_s - 1)\right) + \frac{C_2(R^2 - {r_s}^2)}{2}) \qquad \text{S4}$$

$$\dot{V}_s = 2\pi(\frac{r_s^4}{16\mu_s}(G + \rho_s g) + \frac{C_4 r_s^2}{2}) \qquad \text{S5}$$

The above represents the solution for the cone opening design. For the straight opening design, the final timepoint must be adjusted to also include the additional time it takes for the central sample stream to expand to its full width. Additionally, since the straight opening design only has five elements, it is only compatible with Reaction Classes 2 and 3.

The uncertainty in the timepoint has three main contributors: the transit time through the Kenics insert, the flow dispersion due to the parabolic flow profile in the observation region of the sample cell, and the travel time through the vertical height of the X-ray beam ("beam smearing"). Here, we approximate the uncertainty due to the transit time through the Kenics as half of the average transit time. We added the transit time uncertainty, the flow dispersion, and the beam smearing in quadrature to obtain the full uncertainty for each timepoint, similar to the approach taken in (Plumridge *et al.*, 2018).

S2.4 Additional Flow Conditions

Table S1 Reaction Class 2: One Large Biomacromolecule and One Intermediate Sized Ligand in Cone Opening

Sample A Flow Rate (µL/min)	Sample B Flow Rate (µL/min)	Sheath Flow Rate (µL/min)	Timepoint (ms +/- uncertainty)	Distance from Tip (µm)
60	60	203.1	10 +/- 6	243
60	60	203.1	20 +/- 6	655
30	30	101.5	20 +/- 12	242
30	30	101.5	50 +/- 12	867
30	30	101.5	100 +/- 13	1899
20	20	67.7	100 +/- 18	1210
20	20	67.7	250 +/- 23	3297
10	10	33.8	250 +/- 38	1566
10	10	33.8	500 +/- 46	3295
10	10	33.8	1000 +/- 71	6745

Table S2 Reaction Class 3: One Large Biomacromolecule and One Small Sized Ion in Cone Opening

Sample A Flow Rate (µL/min)	Sample B Flow Rate (µL/min)	Sheath Flow Rate (µL/min)	Timepoint (ms +/- uncertainty)	Distance from Tip (µm)
60	60	203.1	10 +/- 6	197
60	60	203.1	20 +/- 6	609
30	30	101.5	20 +/- 12	196
30	30	101.5	50 +/- 12	821
30	30	101.5	100 +/- 13	1853
20	20	67.7	100 +/- 18	1164
20	20	67.7	250 +/- 23	3251
10	10	33.8	250 +/- 38	1520
10	10	33.8	500 +/- 46	3249
10	10	33.8	1000 +/- 71	6699

Table S3 Reaction Class 2: One Large Biomacromolecule and One Intermediate Sized Ligand in Straight Opening

Sample A Flow Rate (µL/min)	Sample B Flow Rate (µL/min)	Sheath Flow Rate (µL/min)	Timepoint (ms +/- uncertainty)	Distance from Tip (µm)
60	60	203.1	10 +/- 6	307
60	60	203.1	20 +/- 6	719
30	30	101.5	20 +/- 12	306
30	30	101.5	50 +/- 12	931
30	30	101.5	100 +/- 13	1963
20	20	67.7	100 +/- 18	1274
20	20	67.7	250 +/- 23	3361
10	10	33.8	250 +/- 38	1630
10	10	33.8	500 +/- 46	3359
10	10	33.8	1000 +/- 71	6809

Table S4 Reaction Class 3: One Large Biomacromolecule and One Small Sized Ion in Straight Opening

Sample A Flow Rate (µL/min)	Sample B Flow Rate (µL/min)	Sheath Flow Rate (µL/min)	Timepoint (ms +/- uncertainty)	Distance from Tip (µm)
60	60	203.1	10 +/- 6	261
60	60	203.1	20 +/- 6	673
30	30	101.5	20 +/- 12	260
30	30	101.5	50 +/- 12	8885
30	30	101.5	100 +/- 13	1917
20	20	67.7	100 +/- 18	1228
20	20	67.7	250 +/- 23	3315
10	10	33.8	250 +/- 38	1584
10	10	33.8	500 +/- 46	3313
10	10	33.8	1000 +/- 71	6763

S2.4 Myoglobin and Azide: Straight Opening Results

Absorbance experiments were repeated with the straight opening Kenics device. Data acquired with 15 mM azide were recorded after the expansion region and showed single exponential behavior, with $k = 2.3 \times 10^3\ M^{-1}s^{-1}$. Additionally, the measured dead times (average of 5.5 ms for both devices), show that the sample was fully mixed before it transited half of the insert, demonstrating the effectiveness of the Kenics mixer design. Additionally, it is important to note that any build-up of debris in the mixer, due to impurities in the sample or lack of pre-filtering, or the introduction of bubbles can impede mixing, which was evident by data acquired that could not be fit by a single exponential (data not shown).

S2.4 Myoglobin and Azide: Derivation of the Formulas to Determine Rate Constants and Dead Time from Absorbance Data

For the reaction between myoglobin and azide, two states contribute to the absorbance: unbound myoglobin and bound myoglobin, referred to here as State A and State B. To calculate the absorbance during the reaction, the concentrations, C_A and C_B, and extinction coefficients, ϵ_A and ϵ_B, for both states must be included. Therefore, for this system the intensity, I, at each position is

$$I = I_0 10^{-(\epsilon_A C_A + \epsilon_B C_B)l}. \qquad \text{S6}$$

Here, l is the path length. Since all the molecules start out in State A, C_B is just the total concentration, C_0, minus the concentration of molecules currently in State A. Therefore, we can rewrite equation S1 as

$$I = I_0 10^{-((\epsilon_A - \epsilon_B)C_A + \epsilon_B C_0)l}. \qquad \text{S7}$$

If this intensity of the reacting sample, $I(t)$, is divided by the intensity of the unreacted myoglobin sample, $I(t=0)$, we get

$$\frac{I(t)}{I(t=0)} = \frac{I_0 10^{-((\epsilon_A - \epsilon_B)C_A + \epsilon_B C_0)l}}{I_0 10^{-\epsilon_A C_0 l}} \qquad \text{S8}$$

After simplification and taking the log, we get

$$\log \frac{I(t)}{I(t=0)} = (\epsilon_A - \epsilon_B)C_0 l (1 - {}^{C_A}/_{C_0}) \qquad \text{S9}$$

which can be converted to absorbance to give

$$Absorbance = -\log \frac{I(t)}{I(t=0)} = (\epsilon_B - \epsilon_A)C_0 l \left(1 - {}^{C_A}/_{C_0}\right) \qquad \text{S10}$$

Since the azide concentration is in great excess of the myoglobin concentration, we can use standard pseudo-first order chemical reaction equations to express C_A/C_0 as an exponential function, $e^{-(t-t_{dead})/\tau}$, where t represents time, t_{dead} is the dead time, or the time between the beginning of the reaction and the beginning of observation, and τ is the time constant of the reaction, which is directly proportional to the rate constant, k. Therefore, we can rewrite Equation S10 as

$$Absorbance = -\log \frac{I(t)}{I(t=0)} = (\epsilon_B - \epsilon_A)C_0 l (1 - e^{-(t-t_{dead})/\tau}) \qquad \text{S11}$$

S2.4 Trypsin and Aprotinin: Assessment of Reproducibility

The reproducibility of measurements made with the chaotic advection mixer was assessed in two ways. In the first test, a 32 ms timepoint was probed, then the mixer was repositioned, and a different timepoint measured. Then, the mixer was returned to its original location so that the first timepoint could be probed again. This test yielded the repeatable profiles shown in Figure S5a. In the second test, both the flow rate and distance along the channel were adjusted to the same timepoint at two different conditions. These profiles also agreed well and are shown in Figure S5b. These tests demonstrate that the mixer produces robust, repeatable results over the course of an experiment and across a range of flow rates. Figure S5c-d show the Kratky plots of the full time series for comparison and to demonstrate that the 32 ms has a distinct profile.

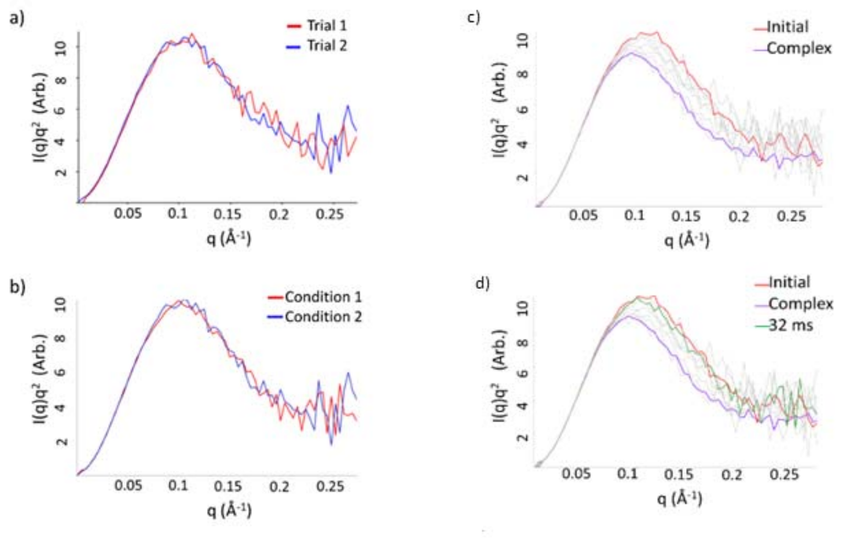

Figure S5. SAXS Kratky profiles were used to assess repeatability. For a)-b), all curves represent a 32 ms timepoint acquired under different flow conditions and should match well. a) Measurement repeated at different times during the experiment with the same flow conditions. b) Measurements acquired using two different sets of flow conditions. c) Shows the Kratky plots of the full time series with the initial and complex states emphasized. d) Shows the Kratky plots of the full time series with the 32 ms and complex states highlighted. The 32 ms profile has specific features not seen in the other timepoints.

S3 SASBDB accession codes

SASDRE3 - Tissue Transglutaminase + Ca: Time-resolved 0ms

SASDRF3 - Tissue Transglutaminase + Ca: Time-resolved 32 ms

SASDRG3 - Tissue Transglutaminase + Ca: Time-resolved 63 ms

SASDRH3 - Tissue Transglutaminase + Ca: Time-resolved 100 ms

SASDRJ3 - Tissue Transglutaminase + Ca: Time-resolved 316 ms

SASDRK3 - Tissue Transglutaminase + Ca: Time-resolved 631 ms

SASDRL3 - Tissue Transglutaminase + Ca: Time-resolved 1500 ms

SASDRM3 - Tissue Transglutaminase + Ca: 5min Equilibrium

SASDRN3 - Tissue Transglutaminase + Ca: 10min Equilibrium

SASDRP3 - Tissue Transglutaminase + Ca: 30min Equilibrium

SASDRQ3 - Trypsin + Aprotinin: Time-Resolved 10 ms

SASDRR3 - Trypsin + Aprotinin: Time-Resolved 30 ms

SASDRS3 - Trypsin + Aprotinin: Time-Resolved 100 ms

SASDRT3 - Trypsin + Aprotinin: Time-Resolved 400 ms

SASDRU3 - Trypsin + Aprotinin: Time-Resolved 630 ms

SASDRV3 - Trypsin + Aprotinin: Time-Resolved 1000 ms

SASDRW3 - Trypsin + Aprotinin: Time-Resolved 2000 ms

SASDRX3 - Trypsin + Aprotinin: Complex equilibrium

SASDRY3 - Kenics GAC rRNA + Mg: Time-Resolved 10 ms

SASDRZ3 - Kenics GAC rRNA + Mg: Time-Resolved 32 ms

SASDR24 - Kenics GAC rRNA + Mg: Time-Resolved 63 ms

SASDR34 - Kenics GAC rRNA + Mg: Time-Resolved 100 ms

SASDR44 - Kenics GAC rRNA + Mg: Time-Resolved 316 ms

SASDR54 - Kenics GAC rRNA + Mg: Time-Resolved 631 ms

SASDR64 - Kenics GAC rRNA + Mg: Time-Resolved 1000 ms

SASDR74 - Kenics GAC rRNA + Mg: Equilibrium initial

SASDR84 - Kenics GAC rRNA + Mg: Equilibrium final

SASDR94 - GAC rRNA + L11 Protein: Time-Resolved 30 ms

SASDRA4 - GAC rRNA + L11 Protein: Time-Resolved 50 ms

SASDRB4 - GAC rRNA + L11 Protein: Time-Resolved 63 ms

SASDRC4 - GAC rRNA + L11 Protein: Time-Resolved 100 ms

SASDRD4 - GAC rRNA + L11 Protein: Time-Resolved 200 ms

SASDRE4 - GAC rRNA + L11 Protein: Time-Resolved 316 ms

SASDRF4 - GAC rRNA + L11 Protein: Time-Resolved 631 ms

SASDRG4 - GAC rRNA + L11 Protein: Time-Resolved 1000 ms

SASDRH4 - GAC rRNA + L11 Protein: Time-Resolved 2000 ms

SASDRJ4 - GAC rRNA + L11 Protein: SEC-SAXS final

SASDRK4 - Diffusive GAC rRNA + Mg: Time-Resolved 10 ms

SASDRL4 - Diffusive GAC rRNA + Mg: Time-Resolved 30 ms

SASDRM4 - Diffusive GAC rRNA + Mg: Time-Resolved 100 ms

SASDRN4 - Diffusive GAC rRNA + Mg: Time-Resolved 300 ms

SASDRP4 - Diffusive GAC rRNA + Mg: Time-Resolved 1000 ms

SASDRQ4 - Diffusive GAC rRNA + Mg: Equilibrium initial

SASDRR4 - Diffusive GAC rRNA + Mg: Equilibrium final

References

Plumridge, A., Katz, A. M., Calvey, G. D., Elber, R., Kirmizialtin, S. & Pollack, L. (2018). *Nucleic Acids Research* **46**, 7354–7365.

Sobiesierski, A., Thomas, R., Buckle, P., Barrow, D. & Smowton, P. M. (2015). *Surface and Interface Analysis* **47**, 1174–1179.

Squires, T. M. & Quake, S. R. (2005). *Rev. Mod. Phys.* **77**, 977–1026.

Wang, Z., Stangegaard, M., Dufva, M., Kutter, J., Wolff, A. & Wang, Z. (2005). *Proc. UTAS 2005* **2**, 745–747.
